# Supplementary material for: Evolutionary Analysis and Functional Identification of Ancient Brassinosteroid Receptors in Ceratopteris richardii
Source: Int J Mol Sci. 2022 Jun 18;23(12):6795. doi: 10.3390/ijms23126795 (PMC9223546; doi:10.3390/ijms23126795)
Supplement: Supplementary file 1 [file ijms-23-06795-s001.zip › ijms-1752706-supplementary.pdf]

Supplementary materials

Evolutionary analysis and functional identification of ancient brassinosteroids receptors in *Ceratopteris richardii*

Bowen Zheng [1], Kaixin Xing [1], Jiaojiao Zhang [1], Hui Liu [1], Khawar Ali [1], Wenjuan Li [1], Qunwei Bai [1] and Hongyan Ren [1,*]

A

	BRI1	BRL1/3	BRL2	BAK1	BAS1	SBI1	BSU1	BIN2	BES1
Angiosperms	+	+	+	+	+	+	+	+	+
Gymnosperms	+	+	+	+	+	+	+	+	+
Monilophytes			+	+	+	+	+	+	+

B

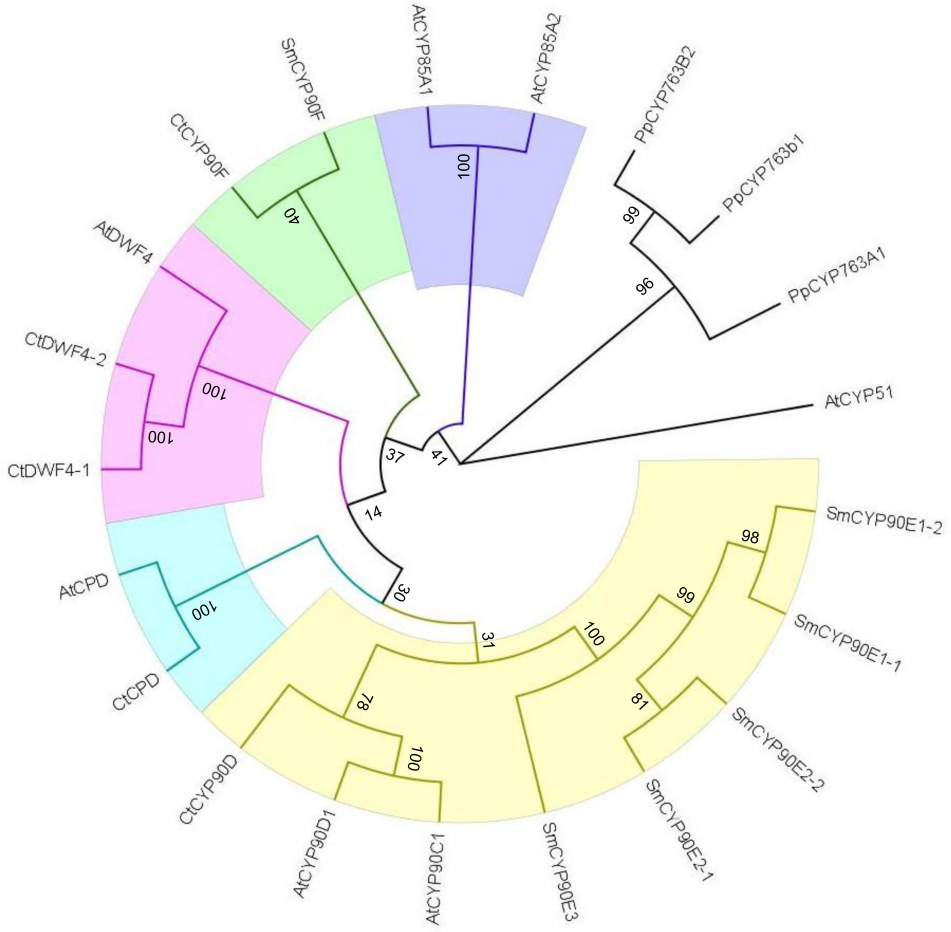

Figure S1. Presence of Key components of the BR signaling pathway exist in *Ceratopteris richardii.* (A) The presence of BR receptors (marked by "+") and main BR signaling components (i.e. BAK1, BSK1, BKI1, BSU1, BIN2 and BES1/BZR1) in plant kingdom obtained by the presence of their transcripts in transcriptomes (B) Phylogenetic tree of the CYP85 family based on the full-length protein sequences. Sequences was aligned using MUSCLE, and the phylogenetic tree was constructed with MEGA7. The tree was generated using the FigTree v.1.4.4. The supporting values were estimated using the SH-Alrt test, and ultrafast bootstrapping with 1000 replicates.

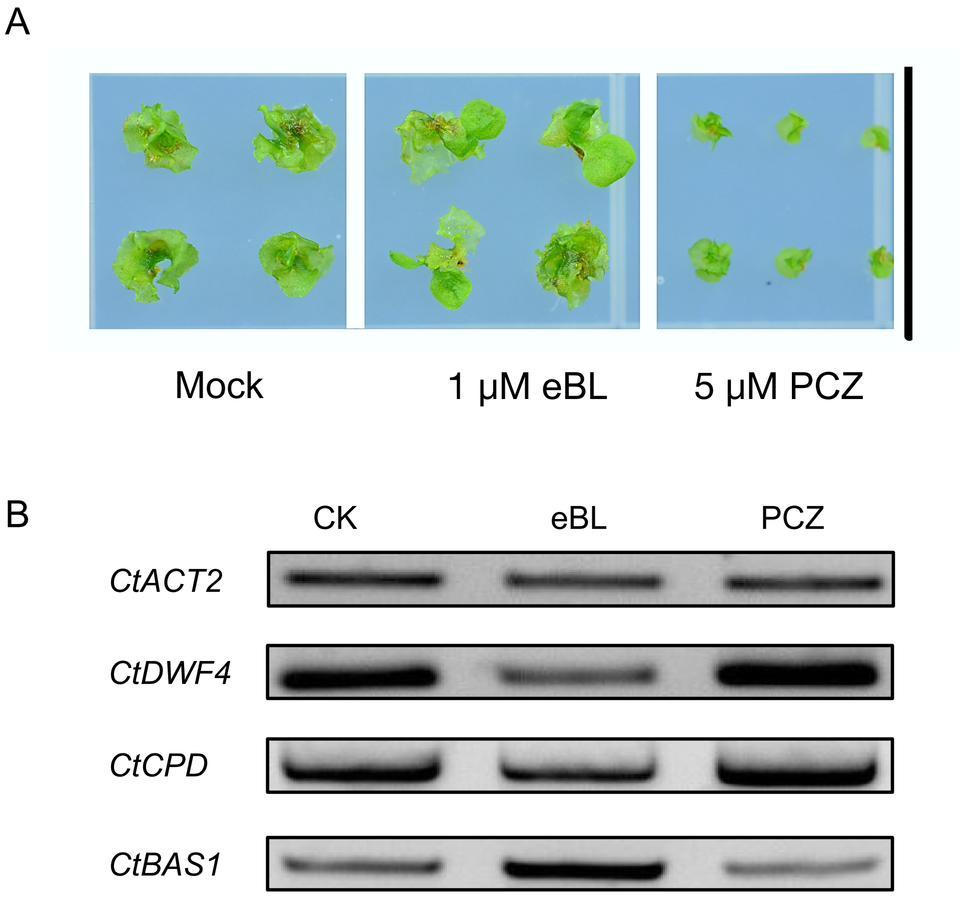

Figure S2. The physiological responses to BRs are conserved in *Ceratopteris richardii*. (A) The *Ceratopteris richardii* grown on 1/2 MS medium with 1 μM eBL or 5 μM PCZ for 50d–old. (B) Semi–quantitative PCR analysis of BR biosynthetic genes *CPD* and *DWF4* and BR catabolic gene *BAS1* in 50–day–old seedlings of *Ceratopteris richardii*, treated with 1 μM or 5 μM PCZ.

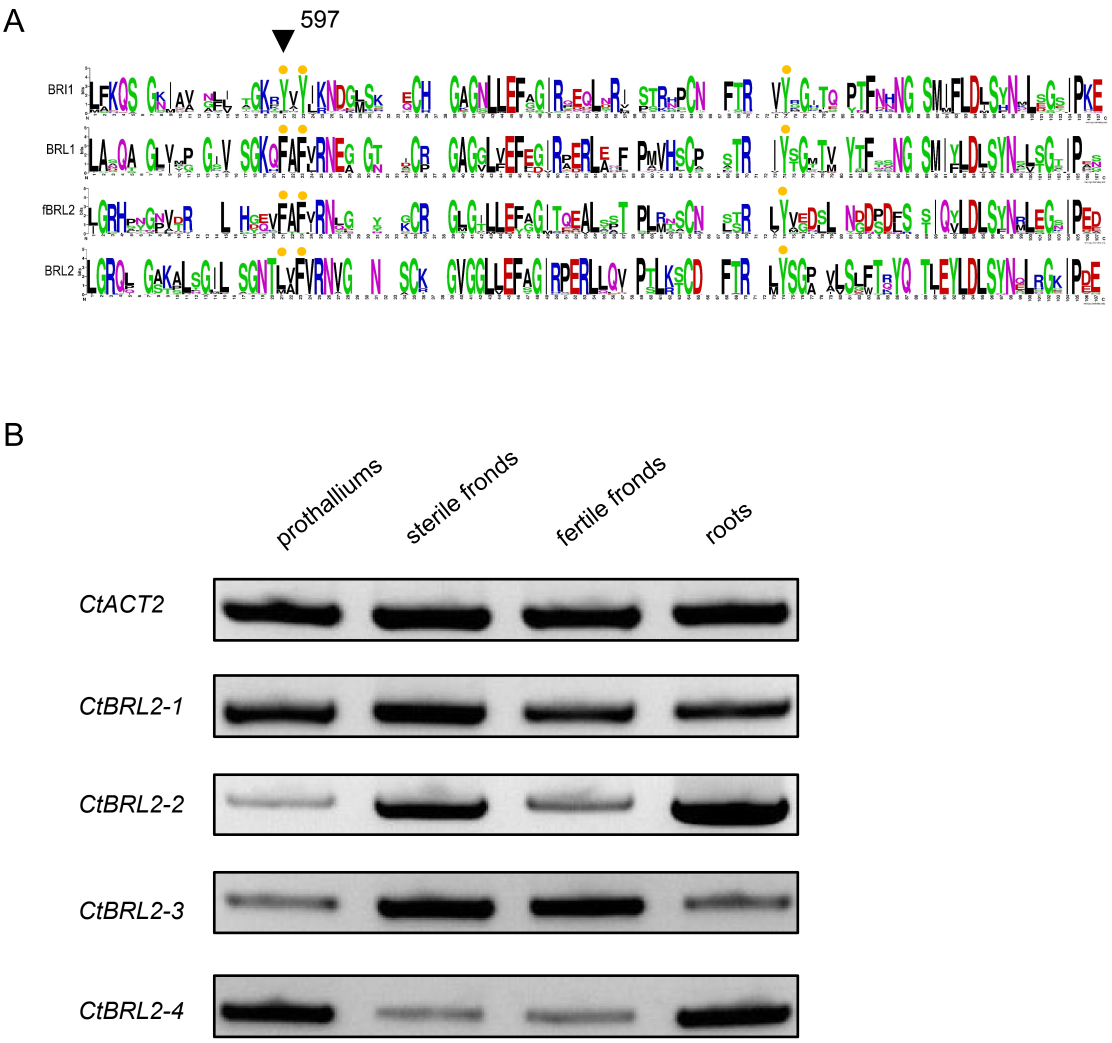

Figure S3. The BL interaction residues of fBRL2 are conserved with AtBRL1. (A) The island domain sequences of BRI1, BRL1 and BRL2 in Angiosperms and fBRL2 in ferns, with the key residues that bind to BL highlighted. (B) Semi-quantitative PCR analysis of BR receptors in different tissues of *Ceratopteris richardii*. A representative image from four biological replicates is shown.

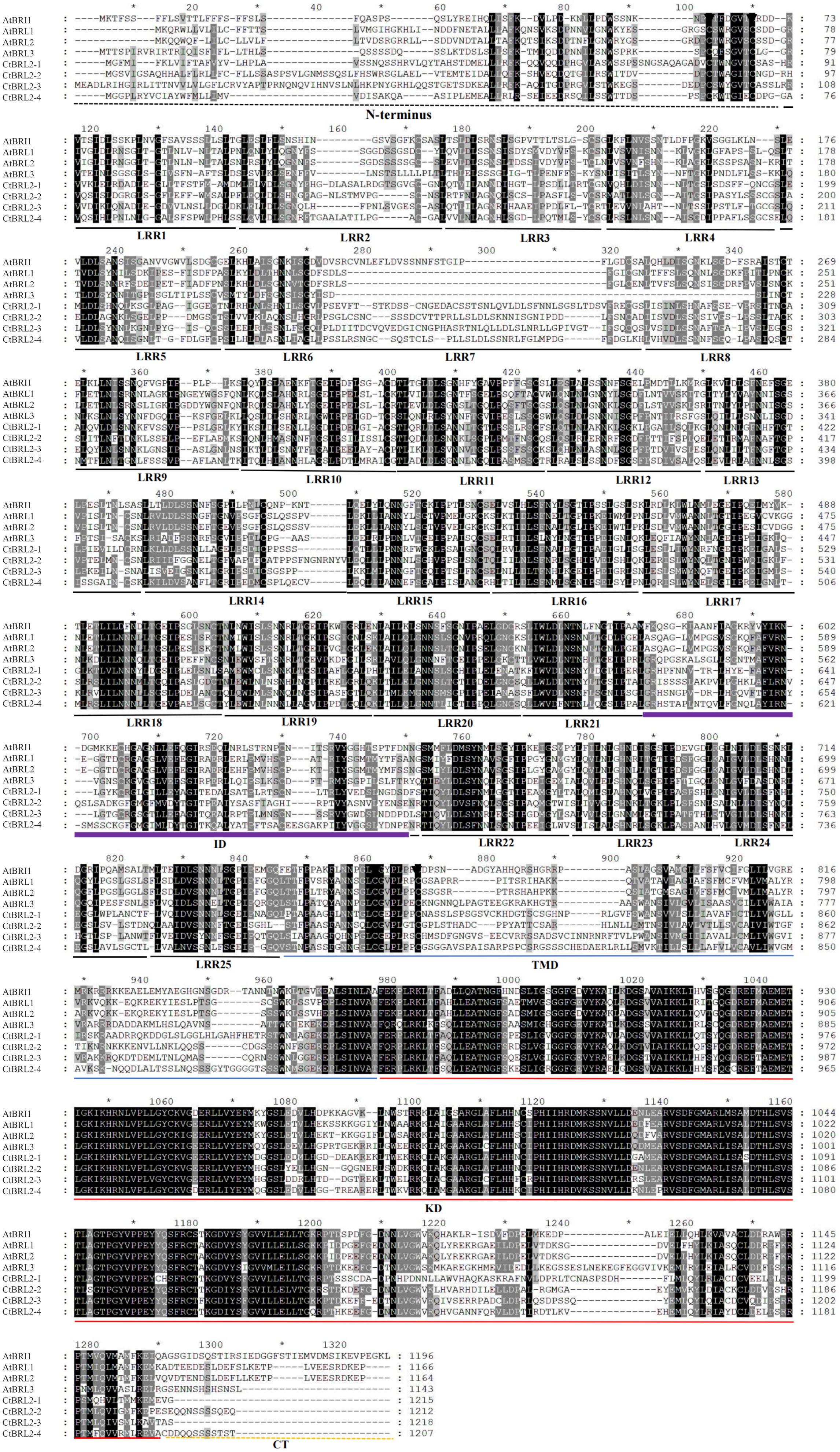

Figure S4. Sequence alignments of four CtBRL2, AtBRI1, AtBRL1/3 and AtBRL2. Conserved residues are highlighted with black. "At" and "Ct" represent *Arabidopsis thaliana* and *Ceratopteris richardii*, respectively.

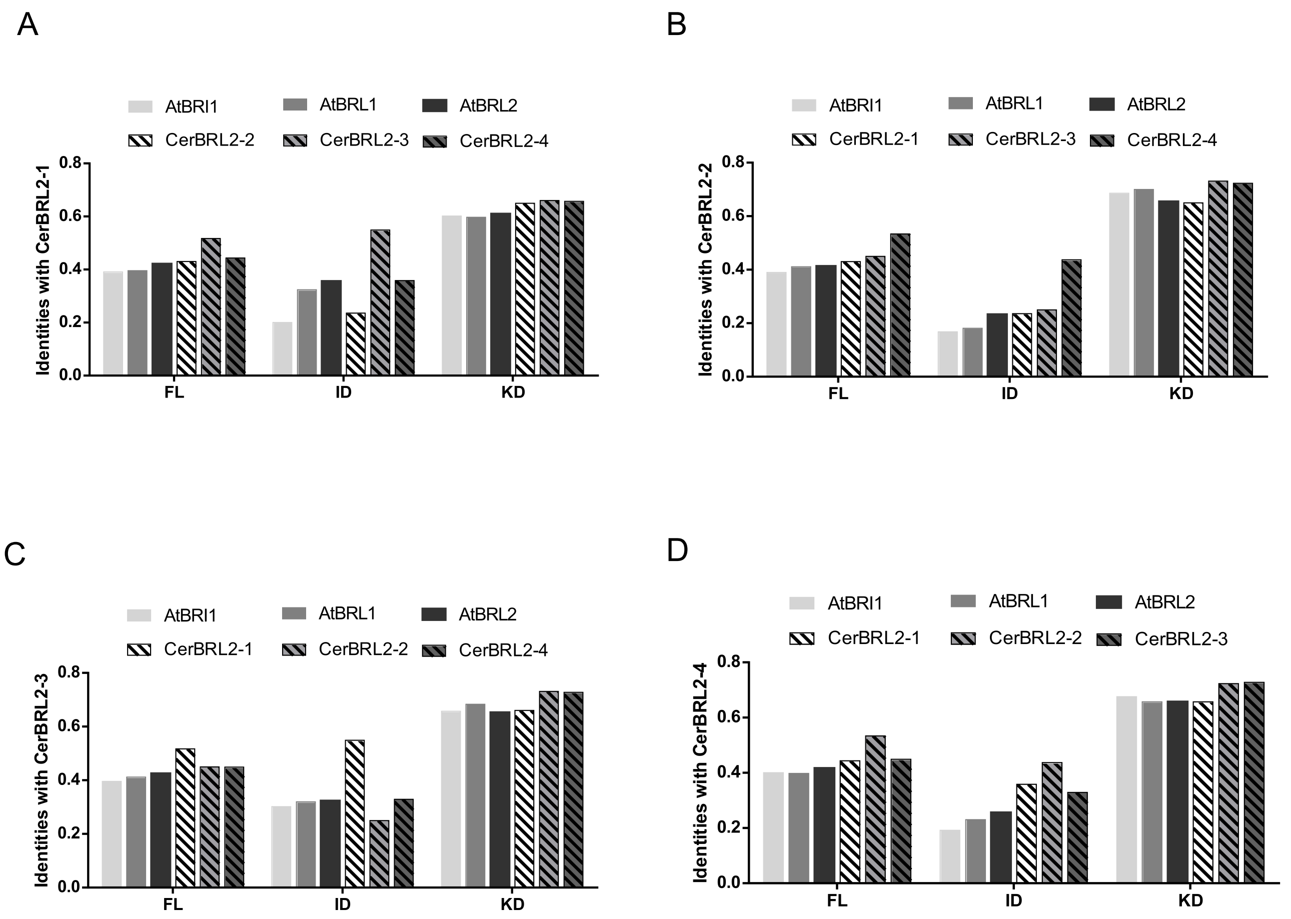

Figure S5. Identity analyses of four CtBRL2, AtBRI1 and AtBRL2. FL, full-length receptors; ID, island domain; KD, Kinase domain. (A) Comparison of CtBRL2-1 with BRI1, BRL1 and other three CtBRL2. (B) Comparison of CtBRL2-2 with BRI1, BRL1 and other three CtBRL2. (C) Comparison of CtBRL2-3 with BRI1, BRL1 and other three CtBRL2. (D) Comparison of CtBRL2-4 with BRI1, BRL1 and other three CtBRL2.

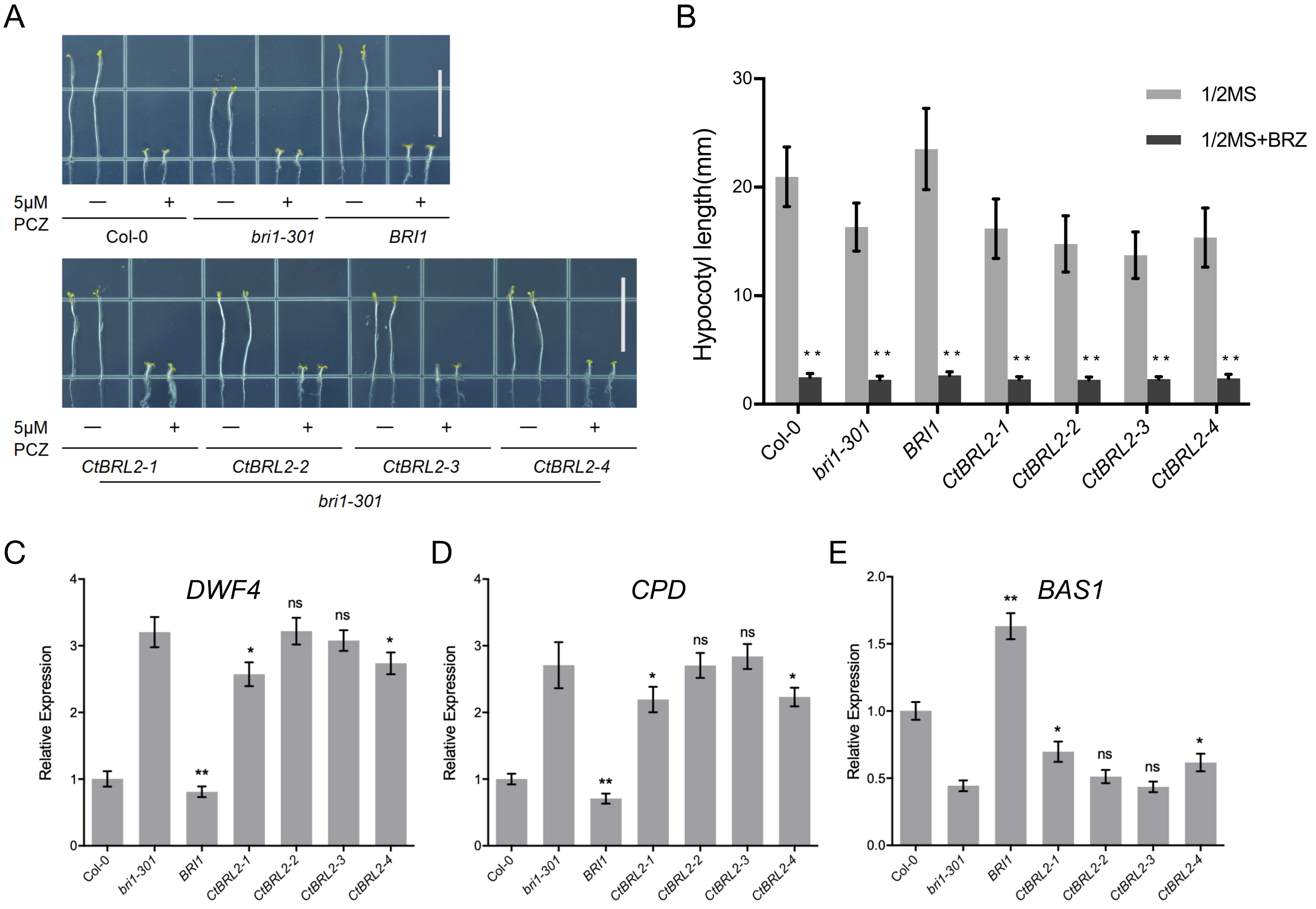

Figure S6. Transgenics expression of different CtBRL2 from *Ceratopteris richardii* does not rescue the phenotype of Arabidopsis *bri1–301* mutants, but does rescue their molecular responses to BR. (A)5–day–old dark–grown seedlings in 1/2 MS medium with or without 5 μM PCZ. Hypocotyl length was plotted as histogram displayed in (B). Scale bar is 1 cm. n = 10 seedlings. **P < 0.01 (two–way ANOVA with Sidak's test). (C–E) Quantitative real–time PCR analysis of BR biosynthetic genes *CPD* and *DWF4* or BR inactivation gene *BAS1* in 4–week–old plants. n = 5 biological replicates. *$P < 0.05$. **$P < 0.01$ (one–way ANOVA with Tukey's test).

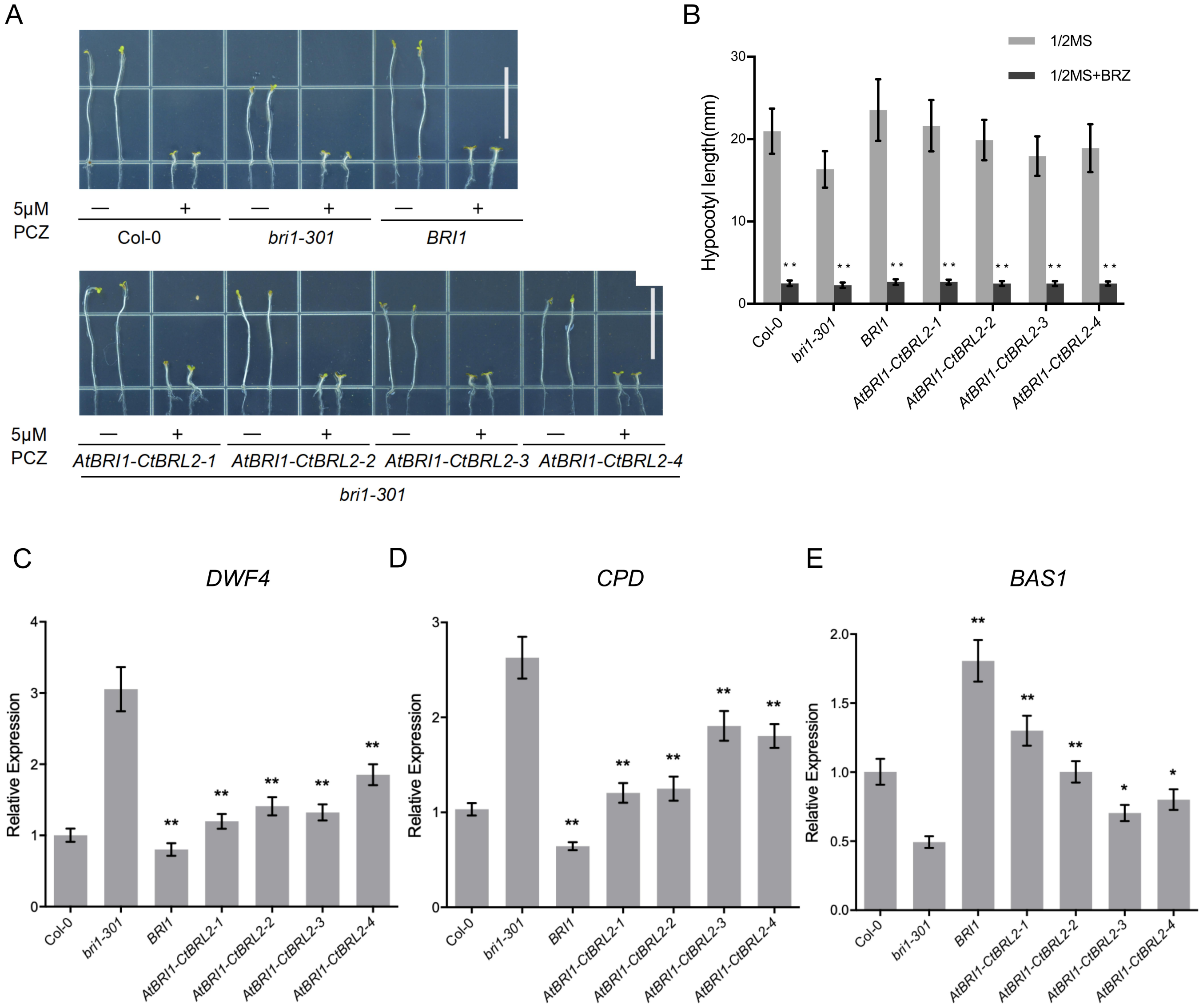

Figure S7. Chimeric receptor *AtBRI1–CtBRLs* can partially rescue the phenotype of *bri1–301* mutants. (A)5–day–old dark–grown seedlings in 1/2 MS medium with or without 5 μM PCZ. Hypocotyl length was plotted as histogram displayed in (B). Scale bar is 1 cm. n = 10 seedlings. **P < 0.01 (two–way ANOVA with Sidak's test). (C–E) Quantitative real–time PCR analysis of BR biosynthetic genes *CPD* and *DWF4* or BR inactivation gene *BAS1* in 4–week–old plants. n = 5 biological replicates. *$P < 0.05$. **$P < 0.01$ (one–way ANOVA with Tukey's test).

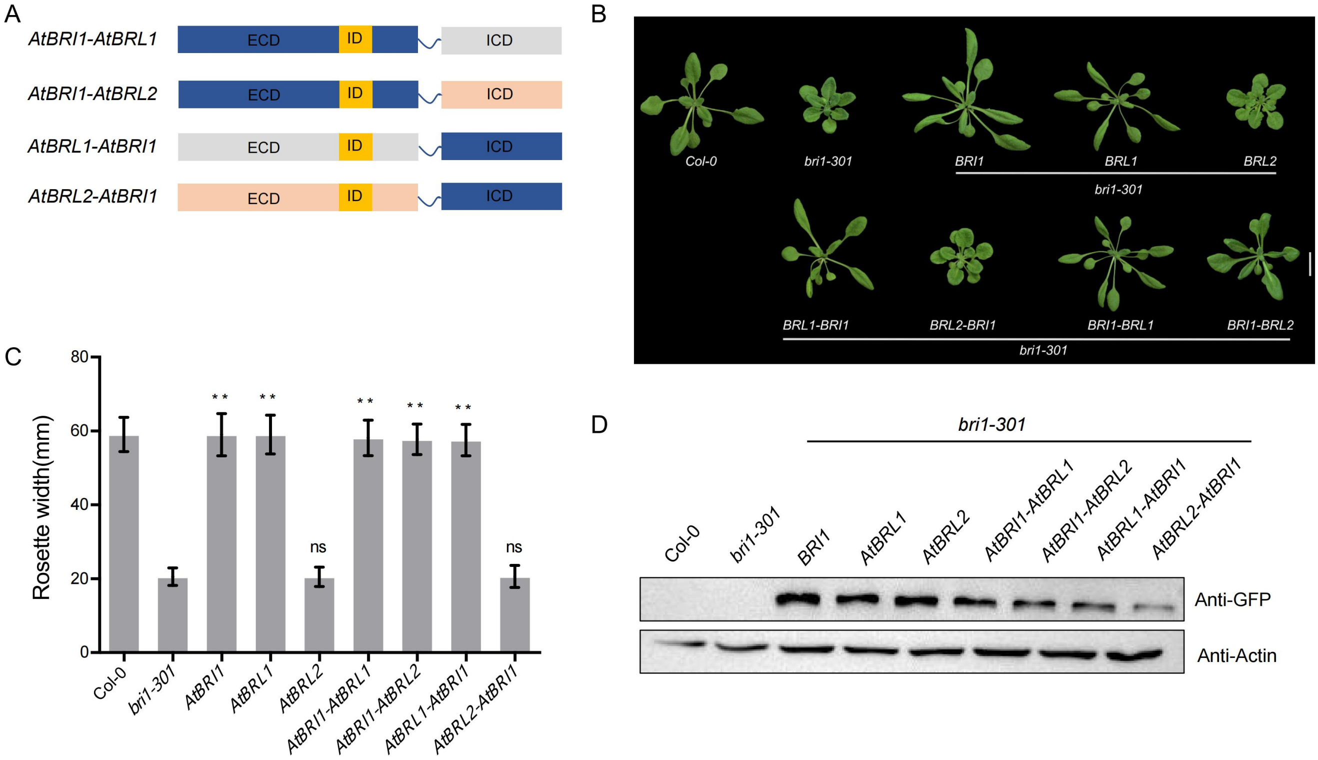

Figure S8. In *Arabidopsis thaliana*, the ICD of BRL1, BRL2, and BRL3, and ECD of BRL1 and BRL3 are interchangeable with BRI1. (A) Schematic diagram of the chimeras *AtBRI1–AtBRL1*, *AtBRI1–AtBRL2*, *AtBRL1–AtBRI1*, and *AtBRL2–AtBRI1.* Different receptor fragments are presented in different colors. (B) Phenotypes of 4-week-old transgenic lines expressing *AtBRI1*, *AtBRL1*, *AtBRL2*, *AtBRI1–AtBRL1*, *AtBRI1–AtBRL2*, *AtBRL1–AtBRI1* and *AtBRL2–AtBRI1* under the *AtBRI1* promoter in *bri1–301* background. The scale bar =1 cm. (C) Quantification of the transgenic lines with the diameter of the rosette leaves in the whole plants grown for 4 weeks, n = 15 plants, $^{**}P < 0.01$, one-way ANOVA with a Tukey's test. (D) Protein expression levels of ectopic genes with GFP tag in the rosette leaves of the transgenic plants as shown in (B). Actin served as the loading control.

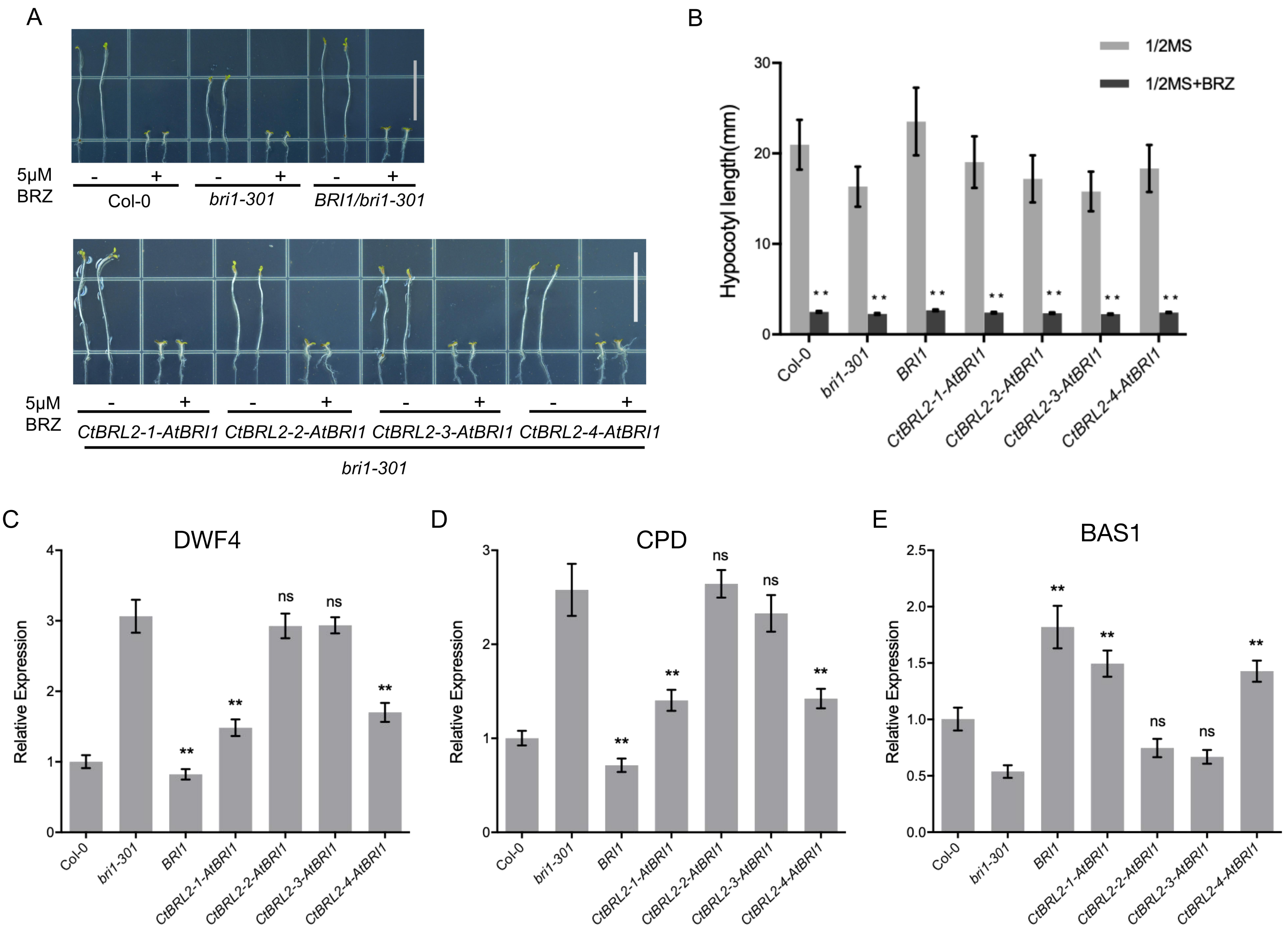

Figure S9. Chimeric receptors *CtBRL2-1-AtBRI1* and *CtBRL2-4-AtBRI1* can partially rescue the dwarf phenotype of *bri1-301* mutants. (A) 5-day-old dark-grown seedlings in 1/2 MS medium with or without 5 μM PCZ. Hypocotyl length was plotted as histogram displayed in (B). Scale bar =1 cm. n = 10. **$P < 0.01$ (two-way ANOVA with Sidak's test). (C-E) Quantitative real-time PCR analysis of BR biosynthetic genes *CPD* and *DWF4* or BR inactivation gene *BAS1* in 4-week-old plants. n = 5 biological replicates. *$P < 0.05$. **$P < 0.01$ (one-way ANOVA with Tukey's test).

A

B

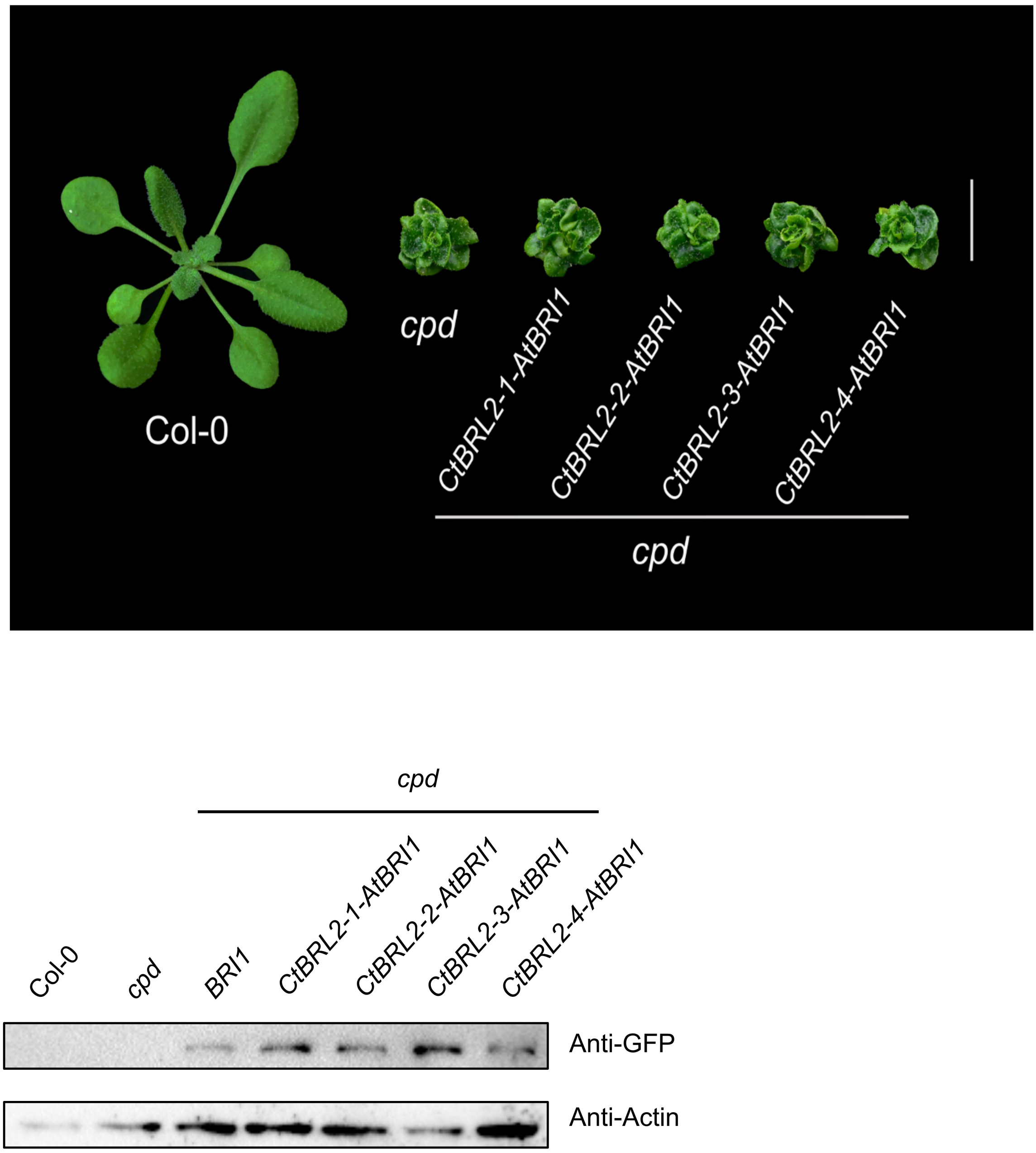

Figure S10. Transgenics expression of four *CtBRL2–AtBRI1* does not rescue the phenotype of Arabidopsis *cpd* mutants. (A) Phenotypes of 4–week–old transgenic lines expressing *CtBRL2–1–AtBRI1*, *CtBRL2–2–AtBRI1*, *CtBRL2–3–AtBRI1* and *CtBRL2–4–AtBRI1* under the control of 35S promoter in *cpd* background. The scale bar is 1 cm. (B) Protein expression levels of the transgenes with GFP tag in the rosette leaves of the corresponding plants shown in (A). Actin served as the loading control.

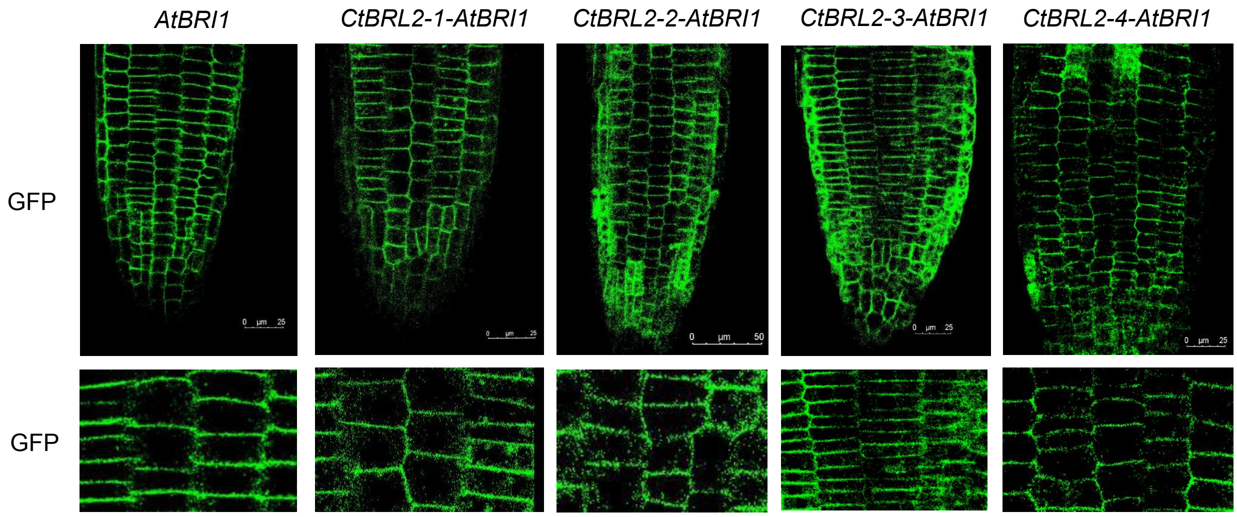

Figure S11. Subcellular localization of the four CtBRL2–AtBRI1–GFP. Confocal images of root epidermal cells of light–grown 4–day–old seedlings showed that all chimeric receptors were localized at the plasma membrane as AtBRI1. Scale bar is 10 μm.

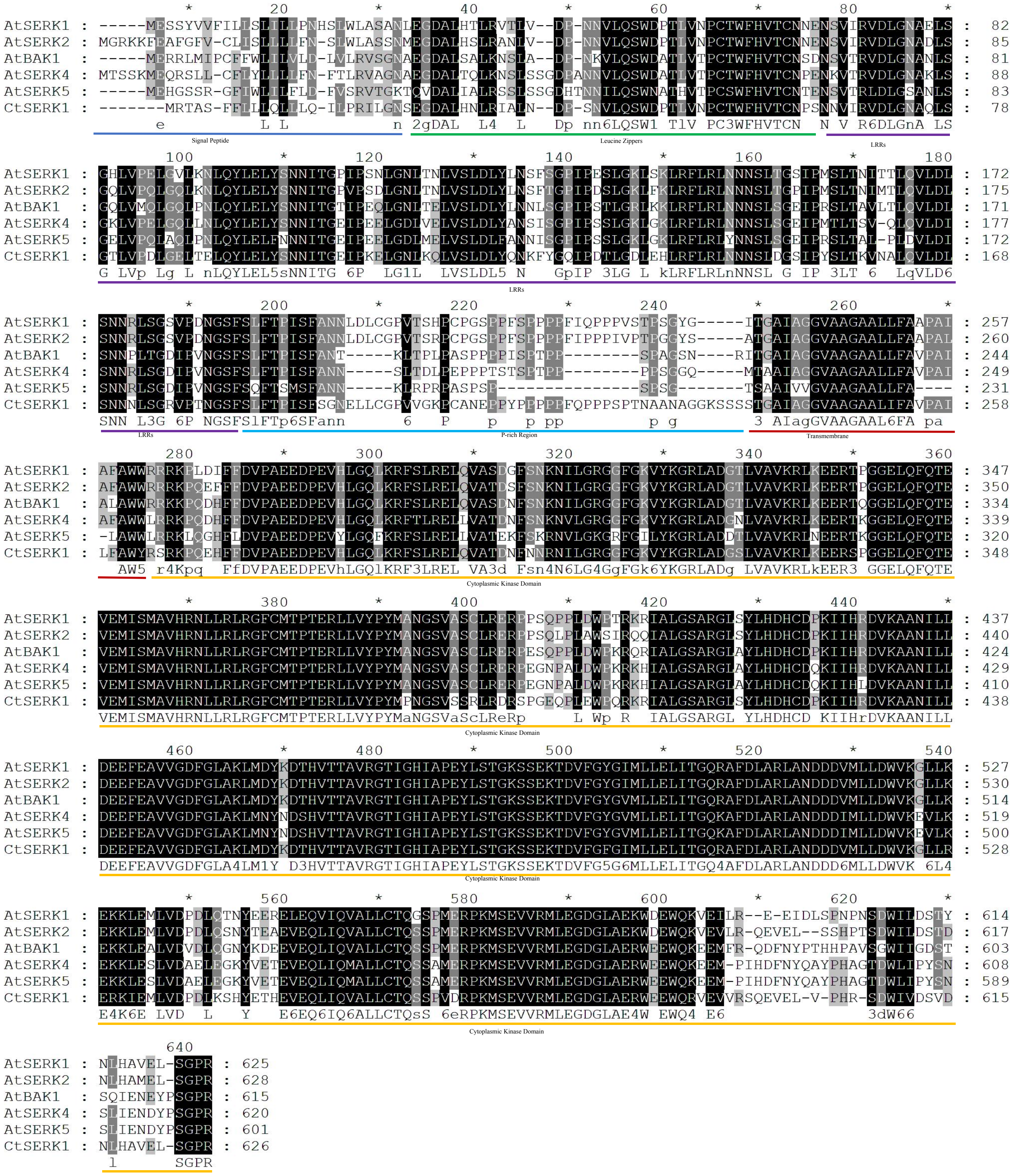

Figure S12. Sequence alignments of CtSERK1, and five SERK family genes in *Arabidopsis thaliana*. Conserved residues are highlighted with black. “At” and “Ct” represent *Arabidopsis thaliana* and *Ceratopteris richardii*, respectively.

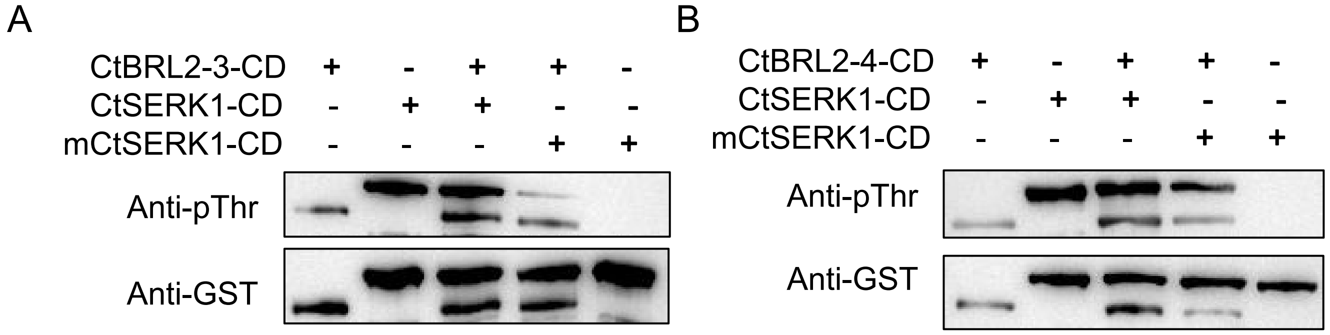

Figure S13. The CtSERK1 works as the co-receptor of CtBRL2s. (A,B) CtSERK1-CD and CtBRL2-CD can both autophosphorylate each other. The kinase assays were performed using CtSERK1-CD-GST and CtBRL2-CD-GST. Top panel, Phosphorylation analyzed by pThr antibody. Bottom panel, the GST served as the loading control.

Table S1 Primers used in cloning, qRT-PCR, and genotyping.

Used for gene cloning	
AtBRI1-F-Kpn1	GCGGTACCATGAAGACTTTTTCAAGCTTCTTTC
AtBRI1-F-BamH1	CGCGGATCCATGAAGACTTTTTCAAGCTTCTTTC
AtBRI1-R-Sal1	GCGGTCGACTAATTTTCCTTCAGGAACTTCTT
AtBRI1-CtBRL2-1-P1	TATTCGCCCACGACGCTGGTCTCCTT
AtBRI1-CtBRL2-1-P2	GGAGACCAGCGTCGTGGGCGAATAGC
AtBRI1-CtBRL2-2-P1	TGTTTGTCATGGACGCTGGTCTCCTTCC
AtBRI1-CtBRL2-2-P2	AAGGAGACCAGCGTCCATGACAAAC
AtBRI1-CtBRL2-3-P1	TACTATTGGCCCACGGCGCTGGTCT
AtBRI1-CtBRL2-3-P2	GGAGACCAGCGCCGTGGGCCAATAGTAT
AtBRI1-CtBRL2-4-P1	TTTTGACCATTGACGCTGGTCTCCTTCC
AtBRI1-CtBRL2-4-P2	AAGGAGACCAGCGTCAATGGTCAAAACC
CtBRL2-1-F-Sma1	TCCCCCGGGCTTGCCATGGGGTTCATGATC
CtBRL2-1-R-Sal1	GTCGTCGACTAAGCCTACCTCCATTTCCTTCAT
CtBRL2-1-AtBRI1-P1	AGCAAGGGAAAACACACCCAATCGCGGGTT
CtBRL2-1-AtBRI1-P2	CGATTGGGTGTGTTTTCCCTTGCTGGT
CtBRL2-2-F-Kpn1	CGGGGTACCATAATCATGGGCTCCGTTATTG
CtBRL2-2-R-Sal1	GTCGTCGACCAATTGTTCTTGACTGCTAGAG
CtBRL2-2-AtBRI1-P1	ACACTACCAGCAAGGGAAAGCAAATT
CtBRL2-2-AtBRI1-P2	TGAATTTGCTTTCCCTTGCTGGTAGTGT
CtBRL2-3-F-Sma1	TCCCCCGGGATGGAAGCGGATCTGAGAATCCATG
CtBRL2-3-R-Sal1	CGTCGACTCTGGAAGCTGTGACTGCCTTTAGC
CtBRL2-3-AtBRI1-P1	AGCAAGGGAGAGCACCGTGAATCGGTTCCT
CtBRL2-3-AtBRI1-P2	GGAACCGATTCACGGTGCTCTCCCT
CtBRL2-4-F-Kpn1	CGGGGTACCCTTTCTCCTCACTGATATG
CtBRL2-4-R-Sal1	GTCCGTCGACTCAAGTACTGGTTGAACTAC
CtBRL2-4-AtBRI1-P1	CTACCAGCAAGGGAGAGGAGACGAAGTC
CtBRL2-4-AtBRI1-P2	GACTTCGTCTCCTCTCCCTTGCTGGTAG
CtSERK1-CD-HIS-F	cagcaaatgggtcgcggatccGCTGCATTGATATTTGCAGTACCT
CtSERK1-CD-HIS-R	tgcggccgcaagcttgtcgacTCTTGGACCTGAAAGCTCAACTG
CtBRL2-1-CD-F	gatctggttccgcgtggatccAAGAGGGCTGCTGATCGAAGG
CtBRL2-1-CD-R	gatgcggccgctcgagtcgacGCCTACCTCCATTTCCTTCATCA
CtBRL2-2-CD-F	gatctggttccgcgtggatccATAAAAAACAGAAACAAGAAGAAGGAGA
CtBRL2-2-CD-R	gatgcggccgctcgagtcgacTTGTTCTTGACTGCTAGAGCTGTTC
CtBRL2-3-CD-F	gatctggttccgcgtggatccGCTGTTGCACTGCTATGCATG
CtBRL2-3-CD-R	gatgcggccgctcgagtcgacGGAAGCTGTGACTGCCTTTAGC
CtBRL2-4-CD-F	gatctggttccgcgtggatccAGCAGCTTGAATCAGTCCTCAAG
CtBRL2-4-CD-R	gatgcggccgctcgagtcgacAGTACTGGTTGAACTACTGCTTTGTTG
AtBRI1-CD-F	gatctggttccgcgtggatccGGTAGAGAGATGAGGAAGAGACGG
AtBRI1-CD-R	gatgcggccgctcgagtcgacTAATTTTCCTTCAGGAACTTCTTTTATAC
CtSERK1-CD-F-Kpn1	acgggggacgagctcggtaccATGCGGACGGCTTCATTTT

CtSERK1-CD-R-Sal1	ttctcctttactcatgtcgacCTATCTTGGACCTGAAAGCTCAACT
mCtSERK1-317-F	GGGTTCGGTGAAGTTTACAAAGGG
mCtSERK1-317-R	CCCTTTGTAAACTTCACCGAACCC
Used for RT and qRT PCR	
CtBRL2-1-RT-F	tgttagaaggagagatccctAAAGAACTAGGGGCTCTTTC
CtBRL2-1-RT-R	atgtttaaacctcttagatcGCTAAAGCTTGCTGGGATAG
CtBRL2-2-RT-F	gatctggttccgcgtggatccCCTCTATCCACGCATGCAGAT
CtBRL2-2-RT-R	gatgcggccgctcgagtcgacGAACATGCCTATGACTTGCAGC
CtBRL2-3-RT-F	tgttagaaggagagatccctAAAGAAATTGGGATGCTCTCC
CtBRL2-3-RT-R	atgtttaaacctcttagatcGGTAAAAGTAGCTGGGATGG
CtBRL2-4-RT-F	tgttagaaggagagatccctAGGGAGCTCGGCAACCTCAC
CtBRL2-4-RT-R	atgtttaaacctcttagatcGGCGAAGCTCGCTGGTAATT
CtBRL2-1-qRT-F	TGATGAGGCGAAGAGGGAGA
CtBRL2-1-qRT-R	AGCTATGGCAGTATTCGGGC
CtBRL2-2-qRT-F	AGAACCCCTGAGCATCAACG
CtBRL2-2-qRT-R	CAAAGCCTCCAATGCCAACC
CtBRL2-3-qRT-F	TTCGGAAGAGTGTGTCAGGC
CtBRL2-3-qRT-R	CATCGGTGTCCTTCTGCCTT
CtBRL2-4-qRT-F	AGAATTTGGAGCCGAGGGTG
CtBRL2-4-qRT-R	TCCAAGATGACGACGCCAAA
CtCPD-qRT-F	CTCGTTTCTGCCCTGGTCAT
CtCPD-qRT-R	GCGCGTTGTGGGGAAAAATA
CtCPD-RT-F	CCAGTTACCCTATGTCGCTTGA
CtCPD-RT-R	GCCCATTCTGAGGGTTTCATT
CtDWF4-qRT-F	TTCTACAGCCCCATTCAGCG
CtDWF4-qRT-R	CATGCGTTTGTGCTCTTCCC
CtDWF4-RT-F	GCGGCCATGGCCATTCTCTTG
CtDWF4-RT-R	GAATCTGTTTGGCCATGAGGT
CtACT2-qRT-F	ATGCTCCAGCCATGTACGTAG
CtACT2-qRT-R	CATAACCCTCATAGATAGGA
CtACT2-RT-F	ATGCTCCAGCCATGTACGTAG
CtACT2-RT-R	TGGCCCAGATTCATCATACTC
CtBAS1-qRT-F	GCAAAAACCATCTTATATTATA
CtBAS1-qRT-R	CACATCAGGAAAATGCTGGTAG
CtBAS1-RT-F	TCTAATGTGGTGCTTGATAAT
CtBAS1-RT-R	GGCAGGAGCGTTAAGGTCACA
AtCPD-qRT-F	GCAATGACGGATGTTGAGAT
AtCPD-qRT-R	CAAGGGTTGAAAGTGCGAGC
AtCPD-RT-F	GTTCTTATCCTGCTTCCATTTG
AtCPD-RT-R	AGCCACTCGTAGCGTCTCATT
AtDWF4-qRT-F	AACAGACGATGATCTTTTGGG
AtDWF4-qRT-R	CTTCAACGGCTTTAGGGCAA
AtDWF4-RT-F	CGAAGGAAGGCTCTTTGAATG

AtDWF4-RT-R	CTTCAACGGCTTTAGGGCAA
AtBAS1-qRT-F	GCCAAATTGACACTCGCTGTAA
AtBAS1-qRT-R	GACGGTAGGTGCATGCTGATAA
AtBAS1-RT-F	GTTCAGGACATTGTGGAGGAG
AtBAS1-RT-R	GGATAAAGCAACATAAGGACG
AtACT2-qRT-F	ACTCTCCCGCTATGTATGTCG
AtACT2-qRT-R	AGAAACCCTCGTAGATTGGC
AtACT2-RT-F	ACTCTCCCGCTATGTATGTCG
AtACT2-RT-R	TGGACCTGCCTCATCATACTC
Used for genotyping	
bri1-116-F	TGGCGAGTTACCGATGGATACG
bri1-116-R	CTCTTAGATCACCTACCTCATCAGG
cpd-F	TTTCTTTCTCTCCGCTCCTTC
cpd-R	CTACTCCGCCGTACACGTTAC
